# Genetic characterization of Lynch syndrome germline variants in a LATAM cohort using a customized NGS gene panel

**DOI:** 10.3389/fonc.2025.1589765

**Published:** 2025-08-01

**Authors:** Cecilia Mathó, Santiago Chávez, Rafael Sebastián Fort, Adriana Della Valle, Florencia Neffa, José Roberto Sotelo-Silveira, Nora Artagaveytia, María Ana Duhagon

**Affiliations:** ^1^ Unidad Académica de Genética, Facultad de Medicina, Universidad de la República, Montevideo, Uruguay; ^2^ Plataforma de Secuenciación Masiva, Instituto de Investigaciones Biológicas Clemente Estable, Montevideo, Uruguay; ^3^ Departamento de Genómica, Instituto de Investigaciones Biológicas Clemente Estable, Montevideo, Uruguay; ^4^ Sección Genómica Funcional, Facultad de Ciencias, Universidad de la República, Montevideo, Uruguay; ^5^ Centro de Oncogenética Uruguayo, Banco de Tumores, Hospital Central de Las Fuerzas Armadas, Montevideo, Uruguay; ^6^ Sección Biología Celular, Facultad de Ciencias, Universidad de la República, Montevideo, Uruguay; ^7^ Departamento Básico de Medicina, Hospital de Clínicas, Universidad de la República, Montevideo, Uruguay

**Keywords:** Lynch Syndrome, colorectal cancer, LATAM, NGS, novel variants, germline cancer predisposition

## Abstract

**Introduction:**

Lynch Syndrome accounts for 1–7% of all colorectal cancers and is caused by germline mutations in DNA mismatch repair (MMR) genes. Timely molecular diagnosis is crucial for effective genetic counseling and management. Among understudied Latin American populations, Uruguay’s genetic admixture provides an opportunity to identify novel Lynch Syndrome related variants.

**Methods:**

This study analyzed 70 unrelated Uruguayan colorectal cancer patients meeting Lynch Syndrome clinical criteria to identify carriers of pathogenic variants. A customized Next-Generation Sequencing (NGS) panel was developed and sequenced on the Ion Torrent platform to analyze nine genes: *MLH1, MSH2, MSH6, EPCAM, FAN1, MUTYH, PMS1, PMS2*, and *APC*. Copy number variations and large EPCAM deletions are not detected by the assay. Gene variants were prioritized based on allelic frequency, *in silico* predictions, pathogenicity records, and ACMG guidelines. The performance of this custom NGS panel was evaluated for in-house applications, and its limitations were thoroughly assessed.

**Results and discussion:**

The custom NGS panel demonstrated effectiveness for scalable in-house testing despite minor disclosed sequence coverage limitations. Pathogenic and likely pathogenic variants were identified in 25 patients, including four novel Lynch Syndrome-associated variants. In four patients, a rare ambiguously classified gene variant co-occurs with a known pathogenic variant in another gene. The mutation profile correlated with clinical parameters such as age of diagnosis, diagnosis criteria, tumor location, and microsatellite instability (MSI).

**Conclusion:**

This is the most comprehensive genetic study to date on a Uruguayan Lynch syndrome cohort. The mutational landscape aligns with findings in other populations while highlighting novel variants of clinical relevance. These findings highlight the value of customized panels for improving genetic screening in small-scale healthcare facilities.

## Introduction

Inherited predisposition to colorectal cancer (CRC) remains a significant concern in cancer care, accounting for 5-10% of new cases/year worldwide (1). Lynch Syndrome (LS) is the most frequent hereditary CRC Syndrome, representing 3-5% of all CRC cases, whereas hereditary polyposis colorectal cancer (HPCC) syndromes account for <1% of CRCs. LS presents an autosomal dominant inheritance and a high risk of developing CRC (8.7-61%) and extracolonic tumors (endometrial, gastric, ovarian, urinary tract, small bowel, pancreatic, brain, or cutaneous cancers) in comparison to the general population (2, 3). Clinical management of probands with a personal or first-degree family history of CRC involves increased screening surveillance, with some differences depending on the gene mutation identified, gender, current age, and familial disease manifestation (age of onset, tumor location).

Clinical criteria for identifying individuals at risk for LS are based on Amsterdam I and II or modified Bethesda guidelines, which rely on personal and family history of cancer and tumor pathological characteristics (4, 5). Germline multigene panel test (MGPT) is indicated for patients with a personal history of LS-related cancer if diagnosed ≤50 years old, if they present a synchronous or metachronous LS-related cancer regardless of age, or if they have a family history of LS or LS-related tumors. Additionally, patients with a personal history of a tumor with a deficient mismatch repair (dMMR) pathway, evidenced by Microsatellite Instability (MSI) are recommended to have an MGPT evaluation for LS and other hereditary cancer syndromes. Also, patients with tumor pathology findings or individuals with a family history compatible with LS can be considered for MGPT. Finally, individuals without a personal history of cancer who meet familial criteria for LS can be evaluated using an MGPT if there is no pathogenic family variant known.

Genetic testing of LS requires sequencing genes involved in the MMR pathway, including *MLH1, MSH2, MSH6, PMS1*, and *PMS2 (* 6, 7). Most deleterious mutations in these genes are heterozygous. Deletions in the *EPCAM* gene, while not directly involved in MMR, lead to the epigenetic silencing of the *MSH2* gene located downstream (8). Biallelic germline mutations in the *MUTYH* gene predispose patients to *MUTYH*-associated polyposis (MAP), which is associated with a lifetime risk of colorectal cancer, typically associated with colonic polyps. However, in some patients, CRC sometimes develops without polyps, mimicking LS (9–11). Non-LS HPCC involves *APC, TP53, STK11, BMPR1A, SMAD4*, and *PTEN* genes.

Next Generation Sequencing (NGS) is the current method for identifying genetically heterogeneous diseases with extensive regions of interest (ROIs) (12). Since the NGS workflow is laborious and time-consuming, and as the size of the target region grows, the complexity and uncertainty of interpretation increase, a focused gene panel is a cost-effective approach for a few genes, compared to a large panel of a whole exome or genome. Therefore, most clinical laboratories offer more proven and less complex NGS assays, such as gene panels, to study inherited predisposition to cancer (12).Whole-exome sequencing (WES) costs have decreased significantly and are now comparable to those of targeted gene panels. However, in facilities that utilize small sequencers or have limited resources, panels remain the preferred option. This preference is due to panels requiring less sequencing capacity and generating smaller datasets, which leads to faster turnaround times and reduced computational demands during analysis. More importantly, panels provide a higher sequencing depth, enhancing the sensitivity and reliability of variant detection while reducing the likelihood of incidental or ambiguous findings. These practical considerations continue to make gene panels a favored choice in many clinical and research contexts, such as the Latin American’s (LATAM). The selection of genes for an NGS-gene test depends on their diagnostic performance, that is, the probability of detecting disease-causing genetic variants in a cohort of patients, which depends on the frequency of the mutations reported in the literature (13). Also necessary is sufficient scientific evidence of the association between an altered genotype and pathology and its implications for managing the disease. The recommended molecular genetic testing for germline mutations causing LS is an MGPT including *MLH1, MSH2, MSH6, and PMS2*, as well as *EPCAM* and other genes of interest that may aid in the differential diagnosis of polypous malignancies (HPCC) and HCRC overlapping syndromes (5, 14)

Herein, we present the genetic testing of 70 Uruguayan LS cancer patients from unrelated families using a custom gene panel and NGS Sequencing with an Ion Torrent sequencer. We analyze the panel’s performance and describe the genetic findings of the cohort. The percentage of mutation carriers and the frequency of mutation/gene are comparable to published cohorts. Custom NGS gene panel sequencing is helpful for the limited number of cases expected in small patient populations, providing a fast local response to patients and their families.

## Methods

### Human subjects

Seventy unrelated patients with clinical suspicion of LS were recruited at the Centro de Oncogenética Uruguayo, Banco de Tumores, Hospital Central de las Fuerzas Armadas, Montevideo, Uruguay. Since this is a national reference center in oncogenetics, patients are referred to from various health institutions (public and private). Pedigree analysis and medical history were gathered during a genetic counseling risk assessment appointment, and the fulfillment of the Amsterdam I, Amsterdam II, or Bethesda genetic risk criteria was analyzed according to the Genetic/Familial High-Risk Assessment: Colorectal, NCCN Clinical Practice Guidelines in Oncology (4).Predictive models such as PREMM5 and MMRpro were not used to select patients. All patients signed an informed consent to enroll in this study, approved by the Ethics Committee of Facultad de Medicina, Universidad de la República (Exp. No 070153­000383­16, UDELAR). Patient recruitment, sample extraction and medical data collection were gathered between 2014 and 2018. The sampling size was defined by funding availability.

### DNA extraction

Genomic DNA was extracted from whole blood using QIAamp DNA Mini Kit (#51304, Qiagen), following manufacturer instructions. DNA quality was verified using a 260/280 nm ratio and quantified using a Qubit Fluorometer with Qubit 1X dsDNA HS (High Sensitivity) Assay Kit (#Q33230).

### NGS targeted sequencing

#### Ion AmpliSeq custom panel

An Ion AmpliSeq custom panel was designed with Ion AmpliSeq Designer to sequence 9 genes: *APC, EPCAM, FAN1, MLH1, MSH2, MSH6, MUTYH, PMS1, PMS2*, as detailed in **Supplementary Table 1**. The gene selection was carried out at the start of the project in 2016, based on the literature available at the time. However, subsequent evidence failed to support the clinical relevance of FAN1 and PMS1 for LS, as discussed in forthcoming sections. The ROI spanned the entire coding sequence of the genes (CDS) and at least five nucleotides from the splicing junctions (exon padding) and the complete 5’ and 3’ untranslated regions (UTRs) of the RefSeq transcripts using RefGene v74. The panel resulted in 229 amplicons distributed in two primer pools of 116 and 113 amplicons. Amplicon size ranges between 132 and 374 bp, with a mean length of 334 bp for both pools. These pools cover 62,51 kb of the human genome. This amplicon set theoretically covered 99,08% of the intended gene sequences (**Supplementary Table 1**). Panel design files are available upon request.

#### Libraries and template preparation

For each patient, 10 ng of genomic DNA was amplified by PCR using the Ion AmpliSeq custom panel described above. Barcoded libraries were constructed using Ion AmpliSeq Library Kit 2.0 (#4475345) and Ion Xpress Barcode Adapters (#4474517). The quality and yield of the libraries were then assessed using Agilent Bioanalyzer (#5067-1504, Agilent Technologies). The libraries were diluted and equimolarly combined to obtain 25 µl of 26 pM for emulsion PCR and enrichment of template-positive ISP particles, which was performed in the Ion One Touch 2 system using Ion PGM Hi-Q View OT2 Kit (#A29900).

#### NGS sequencing

Sequencing was carried out in the Ion Torrent Personal Genome Machine (PGM), using Ion 316 Chip v2 BC (#4488145) with the Ion PGM Hi-Q View Sequencing Kit (#A30044). Each sample was sequenced at a mean depth >180X.

All procedures described above (NGS sequencing) were performed according to the manufacturer’s instructions with Thermo Fisher Scientific reagents unless specified.

#### NGS panel performance evaluation

Binary Alignment Map (BAM) files from the Ion Torrent Server were re-analyzed using multiple open-source tools and in-house scripting. The number of counts per amplicon was obtained using HTSeq (15).

Counts per base were acquired using BamtoCov (16). An in-house script was used to calculate the number of bases with coverage > 50X. Specific commands are available upon request.

**Figure 1:**
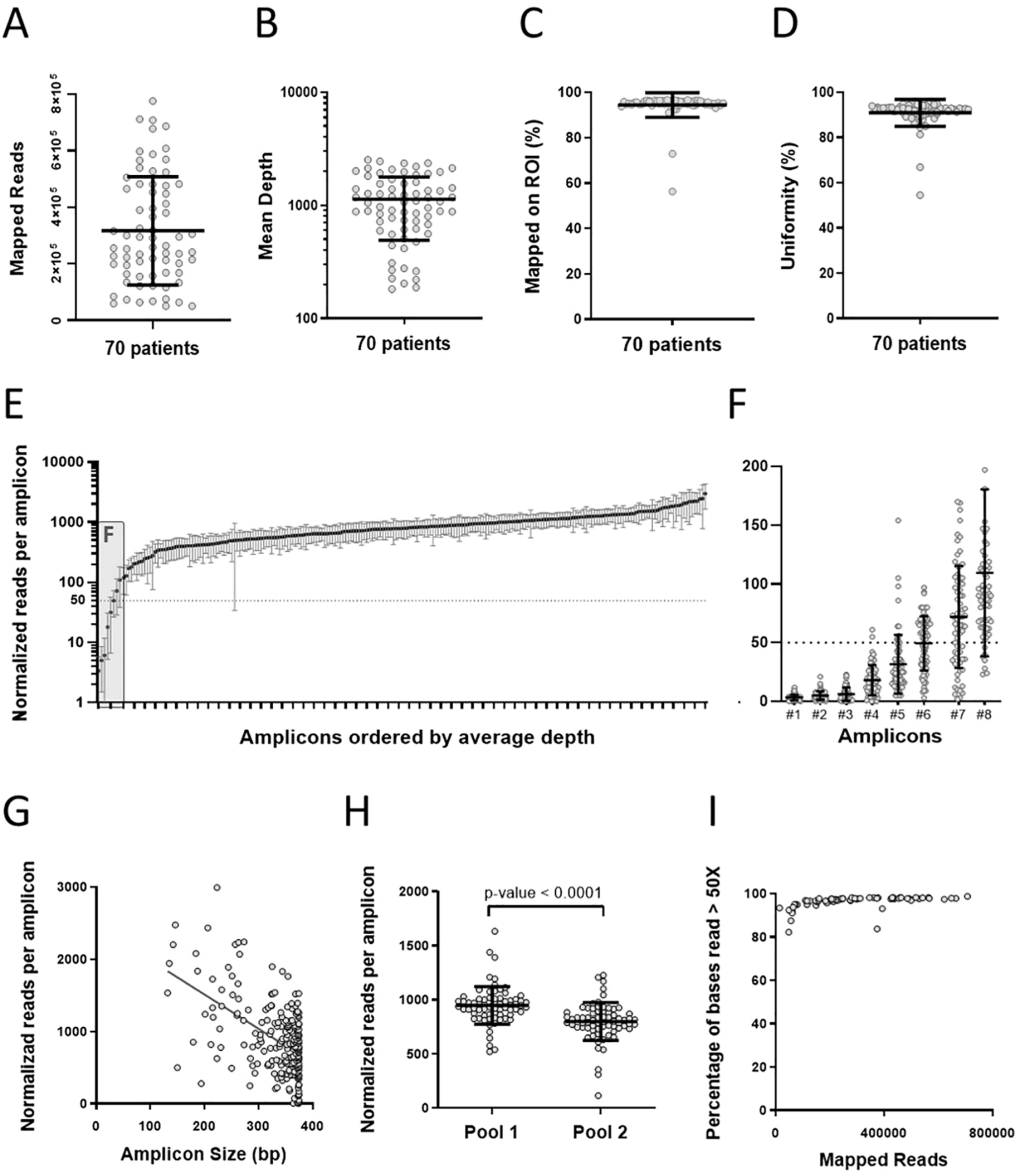
Patient 9-gene panel Sequencing results. **(A–D)** Sequencing metrics per sample. **(A)** Mapped Reads, **(B)** Mean Depth (dotted line indicates 50x depth), **(C)** Reads Mapped on ROI **(D)** Uniformities. **(E–H)** Sequence metrics per amplicon. **(E)** Normalized reads per amplicon ordered by the average depth. **(F)** Detailed view of the first 8 amplicons shown in **(E)**. **(G)** Amplicon performance per size (Slope: -4.74, Spearman’s correlation coefficient: - 0.38, p<0.0001). **(H)** Normalized reads per amplicon pool. The amplicon number difference (116 vs 113) was corrected by normalizing the number of reads to 114.5 amplicons per pool. **(I)** Base coverage analysis: The percentage of bases read more than 50x is plotted against the total mapped reads for each patient. Mean and standard deviations are plotted in all charts. **Supplementary Table 3** contains raw data.

The coverage analysis plugin from the Ion Torrent server was used to obtain the data summarized in **Figure 1** panel A.

#### Genetic variants identification and characterization

After sequencing, the gene variants relative to the Human Genome Reference Consortium Human Build 37 (GRCh37/hg19) were called. A variant call format file (.vcf) was generated using Ion Torrent Server. Each.vcf file was annotated using wANNOVAR (17–19). The variants were prioritized and filtered according to their allelic frequency, zygosity, functional consequence, and database registry. Relevant variants were visually inspected using Integrative Genomics Viewer (IGV) (20, 21) to confirm the read quality.

ClinVar and VarSome’s last accession date was July 18^th^, 2023.

All DNA variants were verified using Version 3.0.8 of Mutalyzer (22), an HGVS compliance tool.

Pathogenic variants detected by NGS were confirmed by Sanger sequencing; reaction conditions are available upon request.

### Tumor microsatellite instability analysis

MSI status was assessed by polymerase chain reaction (PCR), with DNA extracted from paraffin-embedded tumoral tissue and from peripheral blood as previously described by Della Valle et al. as previously described (23). Five markers proposed in the Bethesda panel (BAT25, BAT26, D2S123, D5S346 and D17S250) were amplified by PCR and analyzed by gel electroforesis. Tumors were classified as high (MSI-H) with two or more unstable markers, low (MSI-L) with one unstable marker, or stable (MSS) with no unstable markers.

Immunohistochemistry testing of MMR proteins in tumors was not available in Uruguay when this study was done, so it was not evaluated in this patient cohort.

## Results

### Cohort analysis

This study included 70 unrelated Uruguayan patients with clinical suspicion of LS, whose main clinical characteristics are summarized in **Table 1**. **Supplementary Table 2** provides detailed information. Prior studies have shown that the Uruguayan population comprises ancestral contributions from Europeans, Native Americans, and Africans of consistent proportions among studies (discussed in Bonilla et al, 2015 (24)). According to the largest published study to date, the average ancestral proportions are 76.6 ± 13.7% European, 14.0 ± 10.8% Native American, and 9.4 ± 7.5% African (24). Although patients were referred to by various health institutions, ancestry or sociodemographic biases cannot be ruled out. The median age of onset of the 70 patients and affected family members () was 46 and 58 years old (data not shown), respectively, and the sex distribution was 37 female/33 male patients. The pedigrees of 67 patients were built during consultations with a trained oncogeneticist. Sixty-seven percent of the patients met Bethesda guidelines, while 13% or 20% met Amsterdam I and II criteria, respectively.

**Table 1:** Cohort characteristics.

Number of Patients	70
Sex	Female =37 (53%) Male =33 (47%)
Age of onset	Median =46 years Mean=46,8 years ± 11.5 years (SD)
Diagnostic criteria	Amsterdam I= 9 (13%) Amsterdam II =14 (20%) Bethesda =47 (67%)
Tumor side	Colon Left =19 (27,14%) Colon Right =33 (47,14%) Colon Not specified=6 (8,57%) Rectum only =7 (10,00%), Other location =5 (7,15%)
Tumor Stage	*in situ*= 4 (5.7%) I=1 (1.4%) II = 39(55.7%) III =20 (28,6%) IV = 4 (5,7%) Not Available =2 (2,9%)
Microsatellite Instability	MSI-H=49 (70%) MSI-L=4 (5,71%) MSS=5 (7,15%) Not Available=12 (17,14%)
Tumors in relatives	Colorectal=121 (47%) Breast =40 (15%) Endometrium=5(2%) Ovary= 5 (2%) Others= 87 (34%)
Pathology findings	Differentiation: Poor =1 (1%) Moderate= 27(39%) Well =3 (4%) Not Available =39 (56%)
	Characteristics: Infiltrating = 8 (11%) Ulcerated= 5 (7%) Infiltrating and ulcerated =10 (14%) Infiltrating and ulcerated with mucinous component =1 (1%) Mucinous component= 5 (7%) Signet Ring component =1 (1%) Not Available =40 (57%)

The most common tumor topography among patients was the colon (82.85%) (56.90% right and 32.76% left), followed by the rectum (10%) and other localizations (7.15%), whereas in the relatives was colorectal (47%), followed by breast (15%), endometrium (2%), ovary (2%), and others (34%). More than half of the patients were diagnosed with tumor stage II (55,7%), followed by stage III (28,6%), stage I (1,4%), and carcinoma *in situ* (5,7%). Tumor stage information was unavailable for two patients. MSI analysis resulted in 70% MSI-H, 5.71% MSI-L, and 7.15% MSS, while 17.14% of tumors were unavailable to analyze. Pathology reports were available for 46/70 patients and showed that most tumors were moderately differentiated.

### Sequencing yield and performance of the gene panel

A custom gene panel was used for DNA-seq (**Supplementary Table 1**), and the sequencing results are presented in **Figure 1** and **Supplementary Table 3**. A scatter plot of the number of reads per sample mapped to the reference genome shows an average of 300000 reads/sample (**Figure 1A**). All samples reached the 50x sequencing depth threshold recommended for identifying germline variants by NGS. The average sequencing depth was 1132, and the lowest was 182 (**Figure 1B**). In addition, the proportion of reads mapped to the target sequences exceeds 94% (**Figure 1C**), and the uniformity of DNA sequencing resulted in an average of 90% of the bases read above 20% of the average coverage (**Figure 1D**).

Evaluating the ROI sequence coverage is crucial, as it directly impacts the reliability of our results. The total number of reads per sample was adjusted proportionally to 200,000 reads to normalize the sample sequencing variations. This arbitrary value is close to the median reads obtained per sample, significantly exceeding the expected minimum coverage 50x for each amplicon (**Figure 1E**). Despite most patients being sequenced at a depth greater than 200.000 total reads/sample, 6 amplicons yielded less than 50 reads in the normalized data (**Figure 1F**). The total bp covered by these 6 amplicons is 1944 bp; due to the overlap of other amplicons, this number was reduced to 1888 bp, of which only 955 bp are in coding regions (**Supplementary Table 4** summarizes the poorly covered regions in depth). The missing information on sequence variants of these regions comprises two exons of each of the following genes: *FAN1, MSH6, and MUTYH*. These underperforming amplicons were specified in the individual patients’ test reports.

We tested if amplicon size influenced the read yield (**Figure 1G**), finding that short amplicons tend to produce more reads, which may contribute to the low read number of the 6 underperforming amplicons (371bp average length). We also evaluated the performance of each primer pool and found Amplicon pool 1 performed better than 2 (**Figure 1H**). Finally, a base coverage analysis showed that maximum coverage (~98%) is achieved with approximately 200.000 reads per sample (**Figure 1I**).

### Identification of genetic variants

On average, 66 gene variants were found per patient, of which 16 are localized in exons (data not shown). These variants were prioritized and filtered according to their allelic frequency, zygosity, location, functional consequence, and database registry (Genome Aggregation Database (25, 26), ClinVar (27–29), Human Gene Mutation Database (HGMD) (30, 31), The International Society for Gastrointestinal Hereditary Tumors (InSiGHT) Database (32, 33)), Disease-relevant nucleotide changes, classified as VUS, Likely Pathogenic, or Pathogenic, are shown in **Table 2**.

**Table 2:** Gene variants identified in the Lynch syndrome cohort.

Variant number	Gene	Gene variant	Predicted protein	dbSNP	ClinVar allele ID	Type of mutation	Consequence	ClinVar classification	InSiGHT		HGMD public version	Variant class (ACMG) with varsome	Zygosity	Number of patients with the variant
									InSiGHT classification	Clinical classification				
1	APC	NG_008481.3(NM_000038.4):c.905G>A	NG_008481.3(NP_000029.2):p.(Arg302Gln)	rs764841552	453853	coding	missense	Uncertain significance​	Not classified	VUS	NA	Uncertain Significance	het	1
2	APC	NG_008481.3(NM_000038.4):c.2522T>G	NG_008481.3(NP_000029.2):p.(Leu841*)	NA	1934976	coding	nonsense	Pathogenic	Not classified	Pathogenic	CM106357	Pathogenic	het	1
3	APC	NG_008481.3(NM_000038.4):c.5194A>T	NG_008481.3(NP_000029.2):p.(Met1732Leu)	rs752065261	617174	coding	missense	Uncertain Significance	NA	NA	NA	Uncertain Significance	het	1
4	FAN1	NG_032946.1(NM_014967.4):c.289delG	NG_032946.1(NP_055782.3):p.(Val97*)	rs1418844422	2002884	coding	nonsense	Pathogenic	NA	NA	NA	Pathogenic	het	1
5	FAN1	NG_032946.1(NM_014967.4): c.2854C>T	NG_032946.1(NP_055782.3):p.(Arg952*)	rs184745027	2409994	coding	nonsense	Uncertain Significance	NA	NA	CM 158612	Likely Pathogenic	het	1
6	MLH1	NG_007109.2(NM_000249.3):c.116 + 1G>C	NA	NA	NA	non coding	splicing	NA	NA	NA	NA	Likely Pathogenic	het	1
7	MLH1	NG_007109.2(NM_000249.3):c.350C>T	NG_007109.2(NP_000240.1):p.(Thr117Met)	rs63750781	32133	coding	missense	Pathogenic	Class 5: pathogenic	Pathogenic	CM960965	Pathogenic	het	2
8	MLH1	NG_007109.2(NM_000249.3):c.589-9_589-6del	NA	rs587779026	231609	non coding	unknown	Conflicting interpretations of pathogenicity​ Uncertain significance(4);Likely benign(2)	Class 3: uncertain	VUS	NA	Uncertain Significance	het	1
9	MLH1	NG_007109.2(NM_000249.3):c.665del	NG_007109.2(NP_000240.1):p.(Asn222Metfs*7)	rs63751286	95781	coding	frameshift	Pathogenic	Class 5: pathogenic	Pathogenic	NA	Pathogenic	het	1
10	MLH1	NG_007109.2(NM_000249.3):c.790 + 1G>A	NA	rs267607789	95830	non coding	splicing	Pathogenic	Class 5: pathogenic	Pathogenic	CS011552	Pathogenic	het	1
11	MLH1	NG_007109.2(NM_000249.3):c.911del	NG_007109.2(NP_000240.1):p.(Asp304Valfs*63)	rs1553647969	473553	coding	frameshift	Pathogenic	Not classified	Likely pathogenic/Pathogenic	NA	Pathogenic	het	1
12	MLH1	NG_007109.2(NM_000249.3):c.1863del	NG_007109.2(NP_000240.1):p.(Met621Ilefs*16)	NA	NA	coding	frameshift	NA	Not classified	Pathogenic	NA	Likely Pathogenic	het	1
13	MSH2	NG_007110.2(NM_000251.2):c.289C>T	NG_007110.2(NP_000242.1):p.(Gln97*)	rs63750970	91054	coding	nonsense	Pathogenic	Class 5: pathogenic	Pathogenic	CM041803	Pathogenic	het	1
14	MSH2	NG_007110.2(NM_000251.2):c.435T>G	NG_007110.2(NP_000242.1):p.(Ile145Met)	rs63750124	96572	coding	missense	Conflicting interpretations of pathogenicity​ Uncertain significance(12);Benign(3);Likely benign(6)	Class 3: uncertain	VUS	CM011416	Likely Benign	het	1
15	MSH2	NG_007110.2(NM_000251.2):c.761A>G	NG_007110.2(NP_000242.1):p.(Asn254Ser)	rs1558462016	616614	coding	missense	Uncertain significance​	Not classified	NA	NA	Likely Benign	het	1
16	MSH2	NG_007110.2(NM_000251.2):c.942 + 3A>T	NA	rs193922376	45242	non coding	splicing	Pathogenic	Class 5: pathogenic	Pathogenic	CS941511	Pathogenic	het	1
17	MSH2	NG_007110.2(NM_000251.2):c.1847C>G	NG_007110.2(NP_000242.1):p.(Pro616Arg)	rs587779965	133093	coding	missense	Conflicting interpretations of pathogenicity​ Uncertain significance(7); Benign (2); Likely benign(3)	Not classified	NA	NA	Uncertain Significance	het	1
18	MSH6	NG_007111.1(NM_000179.2):c.749T>C	NG_007111.1(NP_000170.1):p.(Val250Ala)	rs587781275	150494	coding	missense	Conflicting interpretations of pathogenicity​ Uncertain significance(7); Likely benign(2)	Not classified	VUS	NA	Uncertain Significance	het	1
19	MSH6	NG_007111.1(NM_000179.2):c.1420G>C	NG_007111.1(NP_000170.1):p.(Val474Leu)	rs1558661621	616724	coding	missense	Uncertain significance​	NA	NA	NA	VUS with minor pathogenic evidence	het	1
20	MSH6	NG_007111.1(NM_000179.2):c.1510A>T	NG_007111.1(NP_000170.1):p.(Lys504*)	NA	NA	coding	nonsense	NA	NA	NA	NA	Pathogenic	het	1
21	MUTYH	NG_008189.1(NM_001128425.1):c.536A>G	NG_008189.1(NP_001121897.1):p.(Tyr179Cys)	rs34612342	20332	coding	missense	Pathogenic/Likely pathogenic​	Not classified	Pathogenic	CM020286	Pathogenic	het	2
22	MUTYH	NG_008189.1(NM_001128425.1):c.1187G>A	NG_008189.1(NP_001121897.1):p.(Gly396Asp)	rs36053993	20333	coding	missense	Pathogenic/Likely pathogenic​	Not classified	Pathogenic	CM020287	Pathogenic	hom & het	3
23	MUTYH	NG_008189.1(NM_001128425.1):c.1276C>T	NG_008189.1(NP_001121897.1):p.(Arg426Cys)	rs150792276	50192	coding	missense	Conflicting interpretations of pathogenicity​ Uncertain significance(11);Likely benign(6)	Not classified	VUS	CM053999	VUS with minor pathogenic evidence	het	1
24	PMS1	NG_008648.1(NM_000534.4):c.1363A>G	NG_008648.1(NP_000525.1):p.(Lys455Glu)	rs748046504	NA	coding	missense	NA	NA	NA	NA	Likely benign	het	1
25	PMS2	NG_008466.1(NM_000535.5):c.2174C>T	NG_008466.1(NP_000526.1):p.(Ala725Val)	rs150630090	182697	coding	missense	Uncertain significance​	NA	NA	NA	Likely pathogenic	het	1

Novel variants are highlighted in gray. NA, not available or applicable; het, heterozygous; hom, homozygous.

**Figure 2:**
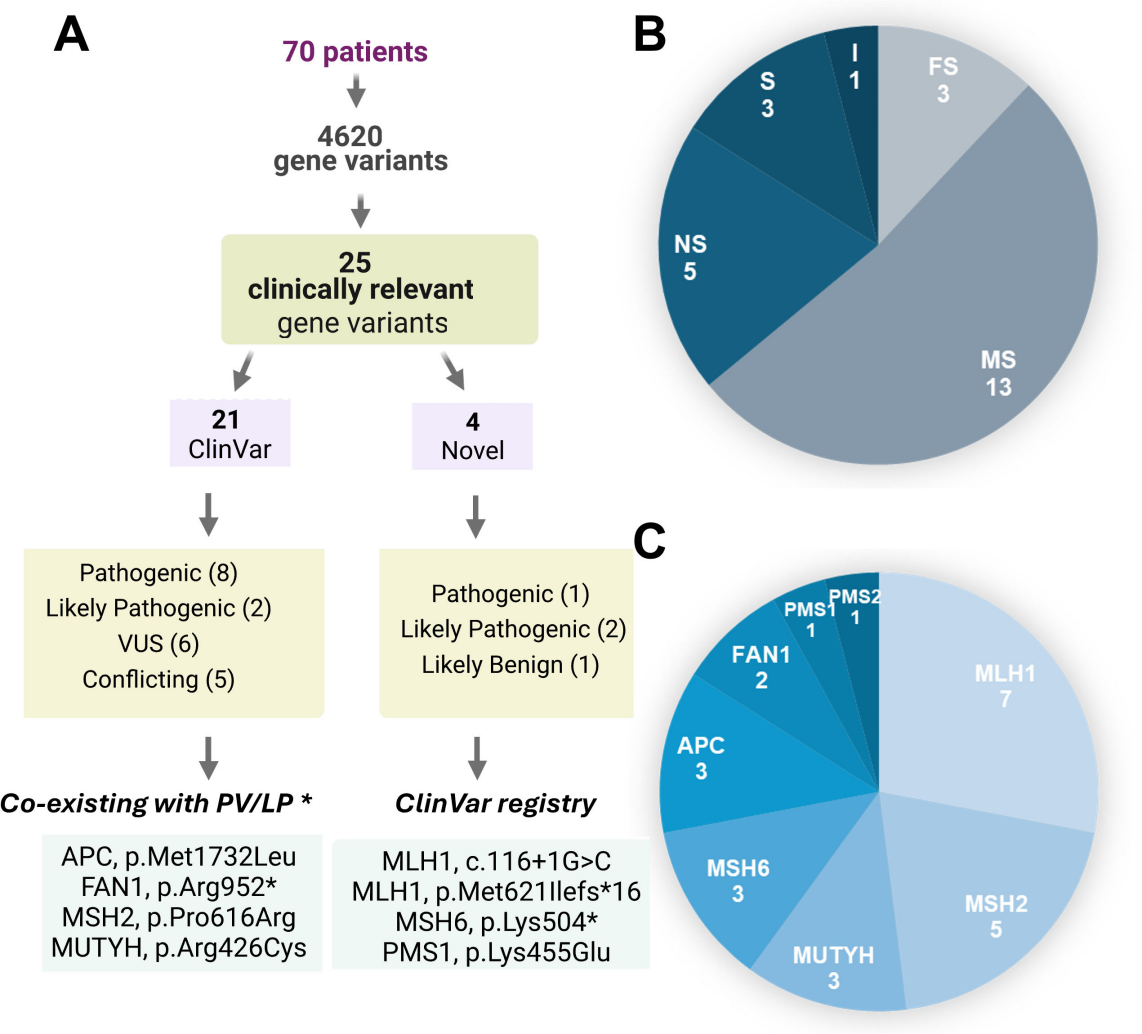
Clinically relevant Variants identified. **(A)** Summary of the variant classification results. *Gene variants coexisting with PV/LP (pathogenic/likely pathogenic variants) in a different gene. **(B)** Distribution of mutation types into MS (missense), NS (nonsense), FS (frameshift), S (splicing) and I (intronic). **(C)** Distribution of mutations in the analyzed genes.

Clinically relevant variants (pathogenic, likely pathogenic, and VUS) were found in 25 patients, representing 35.7% of the cohort. **Figure 2** summarizes the identification and classification of the gene variants in our cohort. The majority (21/25) of the clinically relevant gene variants were classified in ClinVar as follows: Pathogenic (8/25), Likely pathogenic/Pathogenic (2/28), Uncertain Significance (6/25), and another 5/25 resulted in conflicting interpretations of pathogenicity. The remaining 4 were not reported on ClinVar. Following ACMG Guidelines, we have classified them as pathogenic (2), likely pathogenic (1), and likely benign (1). (**Table 3** and **Figure 2A**). Additionally, the coexistence of a potentially clinically relevant variant with a pathogenic variant was observed in four patients (#15, #26, #29, and #39) Patient #15 presents p.Gly396Asp, a biallelic pathogenic *MUTYH* (homozygous), and a p.Arg952* variant in FAN1 (heterozygous). Although this *FAN1* mutation has been previously reported by Segui et al. (46), the association of *FAN1* with LS has been poorly described. Patient #26 is a heterozygous carrier for the pathogenic variant p.Gln97* in MSH2 and variant p.Arg426Cys in MUTYH, which has conflicting interpretations as VUS or likely benign. Patient #29 has the previously described pathogenic variant p.Lys504* in MSH6 and a p.Met1732Leu in APC, already proposed as VUS. Likewise, Patient #39 is heterozygous for the p.Pro616Arg variant in MSH2 (VUS), and the novel variant c.116 + 1G>C in *MLH1* here classified as likely pathogenic. Finally, 4 novel variants were not present in ClinVar, therefore, we submitted them to this database.

**Table 3:** ClinVar novel and VUS-allele frequency and *in silico* predictions.

Variant number (Table 2)	gnomAD AF	SIFT	Polyphen	Mutation taster	Human splicing finder
6	-	NA	NA	DC	Broken WT Donor Site
12	-	NA	NA	DC	New Acceptor splice site. Alteration of auxiliary sequences.
20	-	NA	NA	DC	Alteration of auxiliary sequences
24	0.000006569	D	B	P	No significant impact on splicing signals.
1	0.000006572	D	PD	DC	No significant impact on splicing signals
8	0.00007781	NA	NA	DC	New Acceptor and donor splice sites. Alteration of auxiliary sequences
14	0.0003417	D	PD	P	Alteration of auxiliary sequences
15	–	D	B	DC	Alteration of auxiliary sequences
17	0.00006717	D	PD	DC	New Donor splice site
18	0.00003295	D	B	DC	No significant impact on splicing signals.
19	–	D	PD	DC	No significant impact on splicing signals.
23	0.0008015	D	B	P	Alteration of auxiliary sequences
25	0.00001980	D	B	P	Alteration of auxiliary sequences

GnomAD AF indicates the higher total allele frequency listed. B, benign; CI, Conflicting Interpretations; D, Damaging; DC, Disease-causing; P, Polymorphism; PD, Possibly Damaging; NA, Not available or applicable; -, Not listed on ClinVar.; VUS, Variant of uncertain significance. Novel variants are highlighted in gray.

The gene variants identified were grouped according to type and gene affected (**Figures 2B, C**, respectively). Most are missense variants, followed by nonsense, frameshift, splicing, while an intronic variant at <10 bp of the exon boundary was found. *MLH1, MSH2, MUTYH*, and *MSH6* are the top altered genes, and no variant was identified in EPCAM. Three variants were found in more than one patient: MLH1 p.Thr117Met (2 patients), MUTYH p.Gly396Asp (3 patients: 2 heterozygous and one homozygous), and MUTYH p.Tyr179Cys (2 heterozygous patients).

Further genetic testing with more comprehensive gene panels was performed for seven patients without clinically relevant variants identified using our test; nevertheless, no disease-associated sequence variants were found (**Supplementary Table 2**).

### Analysis of novel variants and VUSes

We analyzed the prevalence and possible phenotypic effects of the four novel variants and the nine already-reported VUSes. *In silico* pathogenicity predictions were performed using Mutation Taster (34), SIFT (35), Polyphen2 (36), and Human Splicing Finder (37). **Table 3** summarizes allele frequencies and *in silico* predictions for novel and VUSes (variants of uncertain significance) reported in ClinVar. Most of the variants were extremely rare or absent in gnomAD, as expected for potentially pathogenic or uncertain variants. For instance, several variants (e.g., 6, 12, 15, 19) were not reported in gnomAD, while others had very low allele frequencies.

*In silico* predictions from SIFT, PolyPhen, and MutationTaster yielded mixed results. While many variants were predicted to be “damaging” (D) by SIFT and “probably damaging” (PD) or “possibly damaging” (P) by PolyPhen (e.g., variants 1, 14, 17), others were predicted as “benign” (B), reflecting uncertainty in their functional impact. MutationTaster frequently classified variants as “disease-causing” (DC), supporting potential pathogenicity. Human Splicing Finder analysis indicated that several variants may disrupt splicing signals. For example, variant 6 was predicted to break the wild-type donor site, while variants 12 and 8 were predicted to create new acceptor and/or donor sites. Multiple variants (e.g., 14, 15, 25) were also predicted to alter auxiliary splicing regulatory elements, suggesting potential effects on mRNA processing. Among the novel variants identified in this study, three of them haven’t been previously reported: so, we classified them using ACMG guidelines, assisted by VarSome (38), and uploaded their information into ClinVar (28) We also uploaded another variant in *MLH1*, not listed in ClinVar (p.Met621Ilefs*16,SCV005329258), identified in a patient who had colorectal cancer at the age of 36, whose father had three surgeries due to three independent colorectal tumors and died aged 54, with no other family members affected to date. This variant was previously reported in a Brazilian family with LS (39).

The patient with the splicing *MLH1* mutation (c.116 + 1G>C,SCV005329268) has 4 affected family members who died without genetic testing. His father died at 64 after rectal cancer, his aunt at 28 years of ovarian cancer, and his grandmother at 72 after uterine, rectum, and ovarian cancer. His grandmother’s sister also had ovarian cancer and died at 39.

The woman with the *MSH6* variant (p.Lys504*, SCV005329267) has 3 dead CRC-affected family members and no genetic testing of other relatives yet. Her father had brain and colon cancer and died aged 37. Two other family members had colon and prostate cancer.

The *PMS1* variant (p.Lys455Glu, SCV005329257) was found in a woman who had colon and rectal cancer aged 70 (MSI-H) and had 4 other affected family members: 3 deceased women with breast cancer (35–50 years old) while her surviving brother had prostate cancer (72 years). To date, none of them have had genetic testing. All the novel and VUS variants identified in this study have an allele frequency that supports the very rare variants causing a high effect, compatible with an inherited cancer predisposition (40). Furthermore, the *PMS1* variant is the only novel variant with a calculated allele frequency in gnomAD, while the others haven’t been found in any allele frequencies’ databases.

During oncogenetic counselling appointments, the probands with pathological variants were informed about the importance of informing at-risk relatives, while clarifying that it is ultimately the proband’s choice whether to disclose. The counsellor also offered to assist in informing relatives with the proband’s permission if communication was difficult, and the consultation and Sanger analysis was offered without cost. Despite the implementation of this counselling strategy, only four relatives pursued genetic screening, resulting in the identification of two carriers of the disease-causing variant.

### Clinical associations

**Figure 3:**
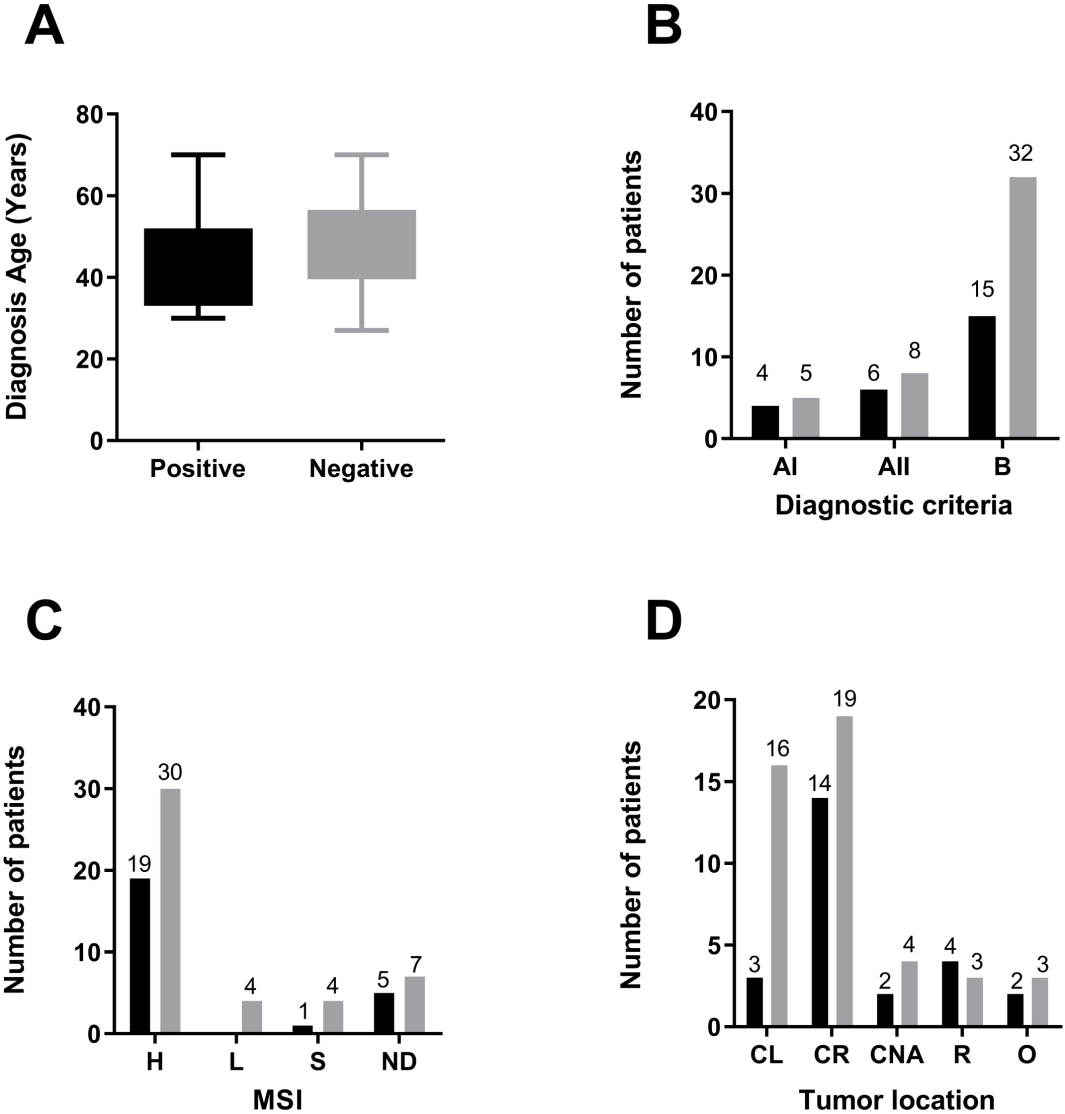
Association between mutations and clinical characteristics of the cohort. Comparison of several aspects among the patients who tested positive for a relevant variant detected (in black), including VUS, likely pathogenic and pathogenic variants, or negative for these variants (in gray). **(A)** Diagnosis Age. **(B)** Fulfillment of Amsterdam Criteria or Bethesda Guidelines. **(C)** MSI status **(D)** Tumor Location. Abbreviations used are CL, colon left; CR, colon right; CNA, colon not assigned; R, rectum; O, other.

To assess the clinical performance of our overall genetic testing, the clinical characteristics of positive and negative results of patients identified by our panel were compared. Since the carriers of disease-predisposing mutations develop cancer at an earlier age than the general population, we compared it among positive and negative patients. The median age of diagnosis is 46 years old, regardless of the mutation status. However, positive patients tend to be diagnosed at a younger age than negative patients, with mean ages of 44 ± 2 and 48 ± 2 years, respectively (Unpaired t-test, two-tailed P value 0.1084, **Figure 3A**).

Genetic assessment of high-risk LS patients involves the use of consensual criteria; thus, patients meeting them have a higher probability of being carriers of disease-predisposing mutations. Overall, the majority of the positive test patients fulfilled Bethesda criteria (15/25), followed by Amsterdam II (6/25) and Amsterdam I (4/25). Of those, 44% of the patients fulfilling Amsterdam I criteria had a positive test result (4/9), followed by Amsterdam II (6/14) and Bethesda (15/47) (**Figure 3B**). High microsatellite instability is associated with LS carrier status due to dMMR. In our study, 70% (49/70) of the tumors evidenced MSI-H; a relevant variant was found in 38.7% of the patients with these tumors. Seventeen percent (12/70) of tumors were unavailable for MSI determination (**Figure 3C**).

Tumor location has also been associated with a genetic predisposition to LS. Patients with right colonic tumors had the highest proportion of positive test results (14/33), and when compared with the left location, we found a significant difference in mutation status supporting the higher occurrence of right colon tumors in carrier individuals (**Figure 3D**).

## Discussion

Herein, we have shown a custom NGS-gene panel’s performance, which proved to be appropriate, having high coverage and uniformity. Although technical evaluation of custom gene panels is cost-effective for genetic diagnosis facilities, it is poorly discussed in the literature (41, 42). The panel included *APC, EPCAM, FAN1, MLH1, MSH2, MSH6, MUTYH, PMS1*, and *PMS2*. Among these, *MLH1, MSH2, MSH6, PMS1*, and *PMS2* code mismatch repair (MMR) proteins. To differentiate LS from other hereditary colorectal cancer syndromes, we also included *APC* and *MUTYH* to rule out familial adenomatous polyposis (FAP) and MUTYH-associated polyposis (MAP), respectively, as these conditions can present with clinical features overlapping LS (43) and they are the top two most prevalent mutated genes in colorectal polyposis syndromes. When this study began the association of PMS1 and FAN1 with LS was still under investigation, but the literature did not subsequently support it. Therefore, for a small gene panel design, we recommend the study of the five genes currently recommended for surveillance/prevention strategies (NCCN Guidelines Version 4.2024 Lynch Syndrome): MLH1, MSH2, EPCAM, MSH6 and PMS2, as well as APC and MUTYH.

Additionally, most reported *EPCAM* alterations involve large deletions, which this panel cannot detect. Indeed, the *EPCAM* gene, located 15 kb upstream of *MSH2*, is relevant because its deletions can lead to *MSH2* inactivation (14, 44–46). Notably, one of the patients with a negative result (Patient #6) was later tested using a commercial panel that detects copy number variations (CNVs). This test identified a deletion encompassing exons 8 and 9 of *EPCAM* and exon 1 of *MSH2*. Although our 9-gene panel was not designed to evaluate CNVs, we assessed *in silico* tools to explore its potential for CNV prediction, confirming that it is not feasible with the amplicons encompassing this gene panel (data not shown). To address this shortcoming, we recommend future gene panels to be specifically designed with optimized amplicon distribution to support CNV detection, provided the assay is well validated for sensitivity and specificity. Rare ambiguous calls might need orthogonal confirmation. Disease-causing CNVs are present in approximately 10% to 20% of Lynch syndrome (LS) cases, depending on the clinical and molecular criteria used to define patient cohorts. Among the mismatch repair genes, MSH2 is most frequently affected by deletions, while large deletions or duplications in MSH6 and MLH1 are observed less commonly (47).

Lastly, *FAN*1 was included in this panel based on a 2015 study by Seguí et al. (48), which initially suggested an association between *FAN1* and colorectal cancer. However, subsequent studies failed to replicate this link, making *FAN1* variant interpretation and patient counseling challenging due to insufficient supporting evidence. Based on our findings, we recommend including *APC* and *MUTYH* in LS syndrome gene panels, as many patients harbor pathogenic variants in these genes despite lacking the typical polyposis phenotype. Conversely, sequencing *FAN1* has not been shown to yield clinically meaningful insights in LS, so we do not recommend its inclusion in future gene panel designs. This is the largest and most comprehensive genetic study of LS patients performed in Uruguay. Previously, LS gene variants of Uruguayan patients using NGS-panels used heterogeneous assays and were mostly outsourced (49). Our study of 70 unrelated patients clinically suspicious for LS identified relevant gene variants in 25 patients (35.7%); although this proportion varies according to the patient selection criteria, it is similar to that of other published cohorts (50–54). Likewise, mutation frequency was similar to other cohorts, with *MLH1* and *MSH2* leading in number, followed by *MSH6 (* 55).

In this study, we report 4 novel variants: *MLH1* (c.116 + 1 G>C), MLH1 (p.Met621Ilefs*16), MSH6 (p.Lys504*), and PMS1 (p.Lys455Glu). The splicing *MLH1* variant is predicted by Human Splicing Finder to disrupt the wild-type donor site. Applying the ACMG criteria, we classified it as Likely Pathogenic. In agreement, another nucleotide change in that position is listed in ClinVar as likely pathogenic (Variation ID: 89656). The frameshift MLH1 mutation is predicted to code for a shorter protein (636 instead of 757 aminoacids), if translated, also Human Splicing Finder predicts splicing alterations; following ACMG Guidelines we classified it as Likely Pathogenic. MSH6 p.Lys504* is a novel nonsense mutation coding a 504 amino acid protein (1360 full-length); probably, its mRNA is subject to nonsense-mediated decay. Accordingly, it is predicted to be disease-causing by Mutation Taster, while Human Splicing Finder predicts alteration of auxiliary sequences. Thus, following ACMG guidelines, we classified it as Pathogenic. Finally, since the *PMS1* variant is predicted to be damaging by SIFT, but Polyphen and Mutation Taster classify it as Benign and Polymorphism, respectively, we classify it as Likely Benign.

Interestingly, in four patients we observed a pathogenic/likely pathogenic variant co-exists with a potentially clinically relevant variant in another gene. The latter variants are rare, with allele frequencies ranging between 0.0013 and 0.0000012. The observation of four patients carrying two independent variants of this type among 25 individuals with known pathogenic mutations appears unlikely, given the reported low population frequencies of these variants. However, such frequencies may currently be underestimated due to the underrepresentation of Latin American populations in existing genetic databases (56, 57).If this is the case, these variants may represent neutral polymorphisms and could eventually be reclassified as benign, pending confirmation through larger genetic studies in Latin American cohorts. Nonetheless, in the absence of segregation data, functional validation, or additional tumor phenotype analyses, we cannot rule out the possibility that some of these variants may function as disease modifiers—or, less likely, contribute to digenic inheritance (48).

Regarding genetic counseling, we found 4 monoallelic *MUTYH* mutation carriers that were notified of their status, based on NCCN guidelines (4), which suggest increased screening only for monoallelic carriers with a personal or first-degree family history of colorectal cancer or polyps.

We also reported the VUSes to the patients. Indeed, we disclosed a novel variant located at 6 pb from the *MLH1* intron-exon boundary (variant #6-**Table 2**), which was later classified as likely pathogenic because Nolano et al (58) demonstrated that it leads to exon 8 skipping.

Our study shows the performance of a 9-gene custom NGS panel for LS mutation identification. The in-house design reduced the cost relative to the commercial alternatives while the platform used is scalable, highlighting its suitability for small-scale healthcare facility in the context of a limited patient population, such as the Uruguayan. Relevant variants were identified in 25 of the 70 patients belonging to independent families, assisted in a single oncogenetic center, and tested using the same methodology, leading to their proper clinical management based on international guidelines. We identified four novel LS disease-causing gene variants and recommended the re-classification of four others as benign.

## Data Availability

The original contributions presented in the study are included in the article/**Supplementary Material**. Further inquiries can be directed to the corresponding author/s.
